# RETRACTION: Magnesium Lithospermate B, an Active Extract of Salvia miltiorrhiza, Mediates sGC/cGMP/PKG Translocation in Experimental Vasospasm

**DOI:** 10.1155/bmri/9896401

**Published:** 2026-04-16

**Authors:** BioMed Research International

RETRACTION: C.‐Z. Chang, S.‐C. Wu, and A.‐L. Kwan, “Magnesium Lithospermate B, an Active Extract of Salvia miltiorrhiza, Mediates sGC/cGMP/PKG Translocation in Experimental Vasospasm,” *BioMed Research International* 2014, 272101. https://doi.org/10.1155/2014/272101.

The above article, published online on 02 April 2014 in Wiley Online Library (http://wileyonlinelibrary.com), and its corrigendum (https://doi.org/10.1155/2016/6240750), have been retracted by agreement between the journal’s Editor‐in‐Chief, Yoel A. Klug; and John Wiley & Sons Ltd.

The authors previously published a corrigendum (https://doi.org/10.1155/2016/6240750) to replace images in Figure 2(a) and Figure 4 following a report on PubPeer [1] of concerns of image duplication and rotation between Figures 2(a), 2(b) and 4. However, a third party also reported [1] that images in Figure 1(b) in this article had been published in several other articles by some of the same authors. Each image was reported differently in the various publications.

The authors did not respond to an inquiry and request for original data by the publisher. Therefore, the retraction has been agreed to because the evidence of image duplication fundamentally compromises the editors’ confidence in the results presented. The authors have been informed of the retraction.
